# Arbuscular Mycorrhizal Fungal Mediation of Plant-Plant Interactions in a Marshland Plant Community

**DOI:** 10.1155/2014/923610

**Published:** 2014-02-12

**Authors:** Qian Zhang, Qixiang Sun, Roger T. Koide, Zhenhua Peng, Jinxing Zhou, Xungang Gu, Weidong Gao, Meng Yu

**Affiliations:** ^1^Research Institute of Forestry, Chinese Academy of Forestry, Beijing 100091, China; ^2^Key Laboratory of Tree Breeding and Cultivation, State Forestry Administration, Beijing 100091, China; ^3^Department of Biology, Brigham Young University, Provo, UT 84602, USA; ^4^Institute of Desertification Studies, Chinese Academy of Forestry, Beijing 100091, China; ^5^College of Resource and Environment, Anhui Agricultural University, Hefei 230036, China

## Abstract

Obligate aerobic AMF taxa have high species richness under waterlogged conditions, but their ecological role remains unclear. Here we focused on AM fungal mediation of plant interactions in a marshland plant community. Five cooccurring plant species were chosen for a neighbor removal experiment in which benomyl was used to suppress AMF colonization. A *Phragmites australis* removal experiment was also performed to study its role in promoting AMF colonization by increasing rhizosphere oxygen concentration. Mycorrhizal fungal effects on plant interactions were different for dominant and subdominant plant species. AMF colonization has driven positive neighbor effects for three subdominant plant species including *Kummerowia striata*, *Leonurus artemisia*, and *Ixeris polycephala*. In contrast, AMF colonization enhanced the negative effects of neighbors on the dominant *Conyza canadensis* and had no significant impact on the neighbor interaction to the dominant *Polygonum pubescens*. AM colonization was positively related to oxygen concentration. *P. australis* increased oxygen concentration, enhanced AMF colonization, and was thus indirectly capable of influencing plant interactions. Aerobic AM fungi appear to be ecologically relevant in this wetland ecosystem. They drive positive neighbor interactions for subdominant plant species, effectively increasing plant diversity. We suggest, therefore, that AM fungi may be ecologically important even under waterlogged conditions.

## 1. Introduction

The arbuscular mycorrhiza, which is a mutualistic symbiosis between plants and arbuscular mycorrhizal (AM) fungi, may enhance plant nutrient acquisition, protect host plants from abiotic (e.g., drought) and biotic (e.g., pathogen) stresses, and mediate plant-plant interactions [1]. The majority of ecological studies on arbuscular mycorrhizas have concentrated on the distribution (~80% of land plant species) and the role of AM fungi in terrestrial ecosystems, as the fungi are considered to be obligate aerobes [2, 3]. Waterlogged soils are generally anoxic [4] and plants under such conditions have traditionally been regarded as nonmycorrhizal [5]. However, mycorrhizal colonization has been reported in waterlogged plants [6–9]. For example, AM colonization does not seem to be significantly disrupted during short-term waterlogging events [10]. Although flooding [8] and redox potential values [11] can influence AM spore numbers and mycorrhizal colonization, molecular technique revealed that aquatic plants may harbor as high an AM fungal species richness as terrestrial plants [8]. However, despite the examination of the existence or prevalence of arbuscular mycorrhiza fungi under waterlogged conditions, more information regarding the ecological significance of AMF in this habitat is still very much needed. In this study we focused on arbuscular mycorrhizal fungal mediation of plant-plant interactions under waterlogged conditions.

Plant-plant interactions are important in driving plant population dynamics [12], plant community structure [13], and ecosystem functions [14]. Beside the long concerned resource competition (negative interaction), the so-called “nurse plant” may benefit the performance of neighboring plant (i.e., positive interaction), through the accumulation of nutrients, provision of shade, amelioration of disturbance, or protection from herbivores [15]. The outcome of plant-plant interactions reflects the balance of negative and positive effects acting simultaneously [16], and the inability to predict the nature of species interactions under various environmental contexts is a major gap in our ecological understanding [17]. Plant interactions are known to be influenced by both abiotic (e.g., the stress gradient hypothesis) [18] and biotic (e.g., herbivores and mycorrhizal fungi) [19, 20] factors. Because of their widespread distribution, fungal mediation of plant interactions by mycorrhizal fungi is of increasing interest.

There is a great deal of variation in the magnitude and direction of the effects of mycorrhizal fungi on plant-plant interactions. In a review paper, van der Heijden and Horton [20] proposed that the mycorrhizal network is very important in ameliorating competition in natural ecosystems. Mycorrhizal fungi also tended to reduce plant competition under saline conditions [21] or enhance positive neighbor effects in severe drought [22]. On the other hand, other studies have shown that mycorrhizal fungi increase plant competition [23–27]. For example, mycorrhizal networks were found to amplify size inequality which was originated from intraspecific competition [26, 27].

Although AMF are not generally characterized as being host specific, AMF species can display host preferences [28–31]. The effect of AM mutualism ranges along a mutualism-parasitism continuum depending on plant species, the life history, and ecological conditions [32, 33]. Then AMF alter plant community structure by affecting the relative abundance of plant species and plant-species diversity [34–37]. AMF could promote plant coexistence by increasing the competitive ability of less competitive species [38, 39] or reduce coexistence by reinforcing competitive dominance of the dominant plant species [40]. In wetland systems, Wolfe et al. [41] and Daleo et al. [42, 43] show that marsh plant zonation and community structure may be dependent on mycorrhizal fungi in these wetland systems. But we still need more studies to obtain a better understanding on mycorrhizal effects in wetland systems.

Here we chose five cooccurring species differing in their competitive ability and environmental optima to evaluate possible AM fungal mediation of neighbor effects in marshland plant community. We also asked whether AM fungal mediation on plant interactions was dependent on oxygen concentration. AM fungal spores are usually abundant in waterlogged ecosystems but fail to develop because of these stressful conditions [8]. A field experiment at the Mar Chiquita coastal lagoon in Argentina demonstrates that fungal colonization is dependent on crab burrowing that can oxygenate soils [43]. Aerenchyma formation in aquatic macrophytes is one of the most obvious adaptive plant responses to flooding [44]. A well-developed aerenchyma in a plant would ensure an efficient exchange of gases between the atmosphere and the soil environment, and some of the oxygen transported through the aerenchyma may leak out of root pores into the surrounding soil [45]. The resulting thick layer of oxygenated soil around individual roots may maintain aerobic microbes. Then these plants may play a “nursing” role on the neighbor individuals with supporting aerobic, beneficial mycorrhizal fungi. Along the Yangtze River in China, marshland is characterized by the dominant plant species, *Phragmites australis*, a large perennial grass commonly found in wetlands. Vegetative organs of *Phragmites australis *have advanced aerenchyma [46], with two field neighbor removal experiments.

We tested the hypothesis that (1) AM fungal symbiosis may show host preference in plant growth promotion and neighbor interaction among the five chosen plant species and (2) *P. australis* existence promotes AM fungal colonization of marsh plant roots by oxygenating waterlogged soils and, in turn, this interaction positively affects the AM fungal mediation of plant interactions.

## 2. Materials and Methods

### 2.1. Site Description

The field experiment was conducted in a freshwater marshland located in Anqing City, Anhui Province (116°59′27′′ E, 30°28′08′′ N), which possesses a subtropical, monsoon climate. Mean annual precipitation is 1500 mm and mean annual temperature is 16.7°C. The site is near the Yangtze River. This marsh typically suffered from immersion from May to the middle of August because of microtides. The vegetation is composed mainly of *Phragmites australis *(Cav.) Trin. ex Steud., *Polygonum pubescens *Blume, *Kummerowia striata *(Thunb.) Schindl., *Leonurus artemisia *(Lour.) S. Y. Hu, *Ixeris polycephala *Cass., and *Conyza canadensis *(Linn.) Cronq.

### 2.2. AMF Mediation of Plant Interactions (Experiment 1)

A field experiment was conducted from April 2012 to August 2012. Five cooccurring plant species differing in their environmental optima and distribution densities were chosen (Table 1). For each plant species, forty 0.5 m × 0.5 m quadrats were established in the field in the middle of April. The 40 quadrats per species were divided into 10 blocks. For each block, quadrats were then randomly assigned to one of the combinations of the following two factors: (1) two levels of AMF: benomyl application versus control and (2) two levels of neighbor treatment: neighbor removal versus neighbor present.

The neighbor removal treatment was used to assess plant-plant interactions by comparing the performance of target plants with or without neighbors [47–49]. At the beginning of the growing season (April), individuals of the same shoot size and number of leaves (within species) were selected for each of the five target plants. Plant pairs were located within 1–3 meters of each other to minimize differences in microclimate but far apart enough to minimize the influence of a nearby pair. After random selection of the target individuals, neighboring plants within the neighbor absent treatment were removed by cutting the aboveground part.

In the benomyl application treatment, the fungicide benomyl (2 g material, with 50% active ingredient, dissolved in 2 L of tap water) was applied to the soil in each quadrat of that treatment to suppress AM colonization [50]. The same amount of tap water without fungicide was added to the control quadrats. Benomyl was not applied since the study site (marsh) suffered from immersion from May to the middle of August, because of microtides and the benomyl cannot be constrained within the treatment quadrats.

Target plants were harvested at the end of the experiment on September 10, after a season's waterlogging. Shoots were separated from roots and were oven-dried at 80°C for 48 h and then weighed. Relative interaction intensity (RII) was used to reflect the nature and strength of plant interactions. RII was calculated as RII = (Xe − Xr)/(Xe + Xr), which is defined as in [51], where Xe and Xr are target biomasses on the existence and removal of neighbors, respectively. RII is defined with limits [−1, +1]. Positive RII values indicate that facilitation prevails; negative RII values indicate that competition prevails.

To evaluate the effectiveness of the fungicide benomyl, roots of the target plants were sampled for testing mycorrhizal colonization. Root colonization by AMF was determined by the gridline intersection method modified by Giovannetti and Mosse [52]. Briefly, roots were cleaned in 10% KOH (w/v), stained in acid fuchsin, and then scored for the presence or absence of mycorrhizal infection (arbuscules, vesicles, coils, or hyphae) under a compound microscope at ×200 magnification. AM colonization level was calculated as AMF colonization (%) = number of intersections colonized (hyphae, arbuscules, vesicles, and hyphal coils)/total number of intersections examined × 100%.

To evaluate possible side effects of the fungicide on soil nutrient and soil microbe [53], soil samples from control and fungicide application plots were taken. Soil nitrogen and phosphorus, enzyme activity, and culturable fungal units were measured (see supporting information for more detailed information in the Supplementary Material available online at http://dx.doi.org/10.1155/2014/923610).

Data of arbuscular mycorrhizal fungal colonization, shoot biomass, and RII were analysed using randomized-block ANOVA, where four treatments in each replicate were blocks. Normality of model residuals and homogeneity of variance between groups were checked for each analysis.

### 2.3. AMF Dependence on Soil Oxygen and the Role of *P. Australis* (Experiment 2)

*I. polycephala *was chosen as model plant here to test whether the effect of AMF was dependent on soil oxygen concentration and whether existence of *P. australis *may increase soil oxygen concentration and promote the role of AMF. Growth of *I. polycephala *was highly dependent on AMF (unpublished data; see also Figure 2), and *I. polycephala *has a high interspecies association with* P. australis* [54]. Another reason for choosing *I. polycephala *is that the vertical distribution of its root system overlaps with that of *P. australis *(both are primarily distributed between 0.1 m and 0.3 m).

To quantify the relationship between the oxygen concentration and mycorrhizal status, soil oxygen concentrations were monitored and root samples of *I. polycephala* were taken from 30 quadrats (1 m × 1 m) randomly placed in the low marsh (nearly inundated daily, c.0.3 m above mean low tide) in September 2012. To collect roots, we excavated the soil to a depth of 0.2 m and transported the whole plant to the laboratory. To avoid the mixture of roots of other plant species, only the roots that were obviously connected to shoots were used to quantify AM fungal colonization. Concentrations of O_2_ were measured by gently pushing a Clark type glass microelectrode (500 *μ*m tip, Unisense A/S Aarhus N, Denmark) into the sediment. The microelectrode was positioned by a micromanipulator and the sensor current was measured with a picoammeter (PA2000, Unisense A/S). The microelectrode was calibrated with both air-saturated and oxygen-free N_2_-saturated water at the same temperature as the sediment.

To quantify the relationship between *P. australis* density and the oxygen concentration, in September 2012, *P. australis* densities were determined within 30 quadrats (1 m × 1 m) randomly placed in the low marsh (almost daily inundated, c.0.3 m above mean low tide). Soil oxygen concentrations were measured as above.

To experimentally test for the effects of *P. australis *on soil oxygen concentration and AMF colonization of *I. polycephala*, a separate field experiment was conducted (April 2012 to September 2012) to evaluate if the presence of *P. australis* affected the oxygen concentration and mycorrhizal status of *I. polycephala*. Twenty 1 m × 1 m random plots were selected in an area of the marsh. In 10 of these plots, *P. australis *was removed by cutting the aboveground in April. After 5 months, the microelectrode was positioned in the center of the plot and near the root of *I. polycephala *to measure oxygen concentration. Roots of *I. Polycephala *were collected from each plot and AMF association was quantified as described previously.

To examine the hypothesis that *P. australis *affects *I. polycephala *growth by affecting mycorrhizal mutualism, a factorial experiment was conducted (from April 2012 to September 2012). Forty 0.5 m × 0.5 m quadrats were established in the field in the middle of April. The 40 quadrats were divided into 10 blocks. For each block, quadrats were then randomly assigned to one of the combinations of the following two factors: (1) two levels of AMF: benomyl application versus control and (2) two levels of neighbor treatment: all neighbors removal versus neighbors removal but with *P. australis *existing. Benomyl application was manipulated as in experiment 1. In all neighbors removal treatment, individual of *I. polycephala *was chosen as target plant, and all the neighbors were removed by cutting the aboveground part, while for the *P. australis *neighbor present treatment, *P. australis *individuals were kept in the plot and all neighbors of the other species were removed, and then the neighbor effect reflected the interaction from the *P. australis *neighbor. Target *I. polycephala *individuals were harvested in September for weighting shoot biomass and measuring AMF colonization.

Linear correlations between oxygen concentration and mycorrhizal status and between *P. australis* density and oxygen concentration were conducted using SAS software (SAS Institute Inc., NC, USA). *t*-test was done for testing *P. australis* removal on soil oxygen and AMF colonization on *I. polycephala. *The GLM procedures were used for comparisons of AMF colonization and shoot biomass of target *I. Polycephala* in the two-factor design.

## 3. Results

### 3.1. AMF Mediation on Plant Interactions (Experiment 1)

#### 3.1.1. AMF Colonization Rate

**Figure 1:**
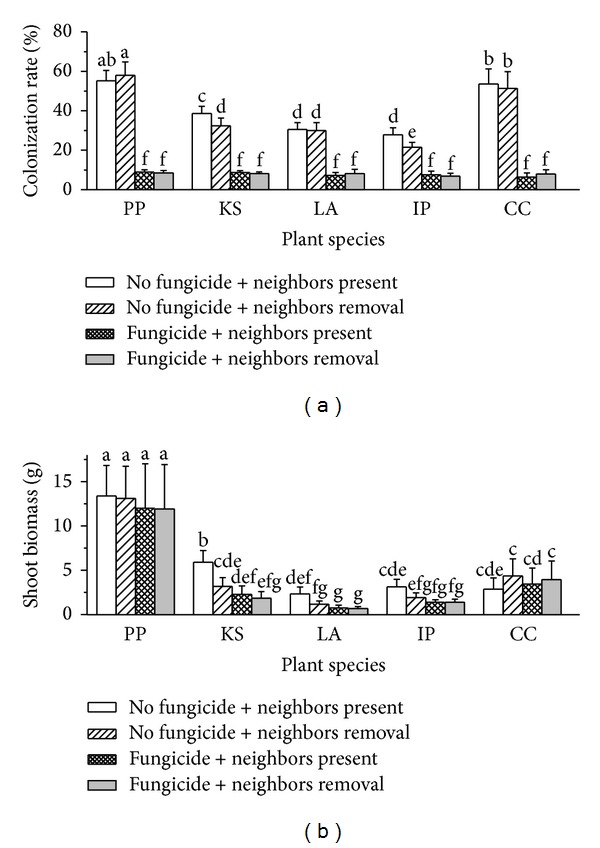
Arbuscular mycorrhizal colonization rate and shoot biomass of target plants under different treatments in the experiment. Data are means ± SD. Different letters represent significant difference among treatments. PP: *Polygonum pubescens*; KS: *Kummerowia striata*; LA: *Leonurus artemisia*; IP: *Ixeris polycephala*; CC: *Conyza canadensis.*

AM fungal colonization rates were significantly different among five plant species (df = 4, *F* = 141.67, and *P* < 0.0001; Figure 1(a)). Benomyl application significantly decreased total AMF colonization of roots (df = 1, *F* = 3579.95, and *P* < 0.0001). Interaction between benomyl application and plant species was significant (df = 4, *F* = 131.22, and *P* < 0.0001; Figure 1(a)). Neighbor removal also affected AMF colonization rate significantly (df = 1, *F* = 4.78, and *P* = 0.031), while this effect was dependent on plant species (df = 4, *F* = 3.25, and *P* = 0.014) and benomyl application (df = 1, *F* = 6.22, and *P* = 0.0138). Interactive effect of plant species, benomyl application and neighbor removal was significant (df = 4, *F* = 2.45, and *P* = 0.049).

#### 3.1.2. Shoot Biomass

Plant shoot biomass was significantly different among five plant species (df = 4, *F* = 303.61, and *P* < 0.0001; Figure 1(b)). Benomyl application decreased shoot biomass significantly (df = 1, *F* = 24.54, and *P* < 0.0001), while this effect was dependent on plant species (df = 4, *F* = 2.98, and *P* = 0.022): for the two dominant plant species *P. pubescens* and *C. canadensis*, fungicide application had no significant effect on plant shoot biomass (Figure 1(b)), and for the three subdominant plant species, fungicide application decreased shoot biomass significantly (Figure 1(b)). Effect of neighbor removal had no significant effect on plant shoot biomass (df = 1, *F* = 2.86, and *P* = 0.093), but its interactive effect with plant species (df = 4, *F* = 3.11, and *P* = 0.018) was significant, showing a species-specific plant response to neighbor removal. Two-way interaction between benomyl application (df = 1, *F* = 2.60, and *P* = 0.109) and three-way interaction of plant species, neighbor removal, and benomyl application (df = 4, *F* = 1.35 m, and *P* = 0.253) were not significant.

#### 3.1.3. Relative Interaction Intensity

**Figure 2:**
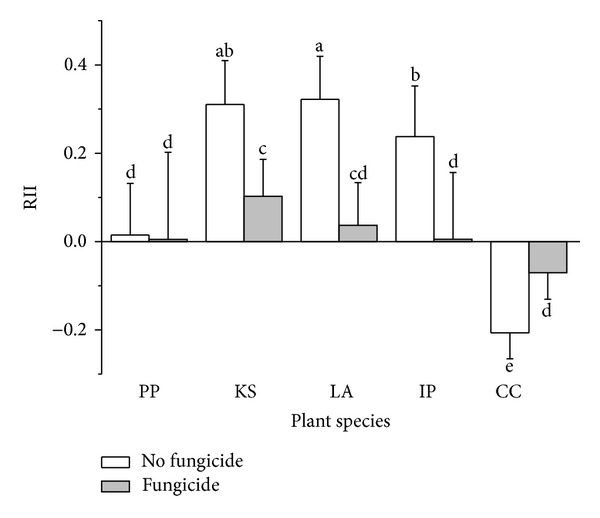
Relative interaction intensity under different treatments in the experiment. Data are means ± SD. Different letters represent significant difference among treatments. PP: *Polygonum pubescens*; KS: *Kummerowia striata*; LA: *Leonurus artemisia*; IP: *Ixeris polycephala*; CC: *Conyza canadensis.*

Plant interaction intensity was significantly different among five plant species (df = 4, *F* = 49.64, and *P* < 0.0001). Benomyl application significantly affected plant interaction intensity (df = 1, *F* = 44.39, and *P* < 0.0001). Interaction between plant species and benomyl application was significant (df = 4, *F* = 19.38, and *P* < 0.0001). Benomyl application decreased positive neighbor effect on the three subdominant plant species, *Kummerowia striata*, *Leonurus artemisia*, and *Ixeris polycephala*, increased negative neighbor effect on *Conyza canadensis*, and had no significant effect on the neighbor effect of *P. pubescens* (Figure 2).

### 3.2. AMF Dependence on Soil Oxygen and the Role of *P. australis* (Experiment 2)

**Figure 3:**
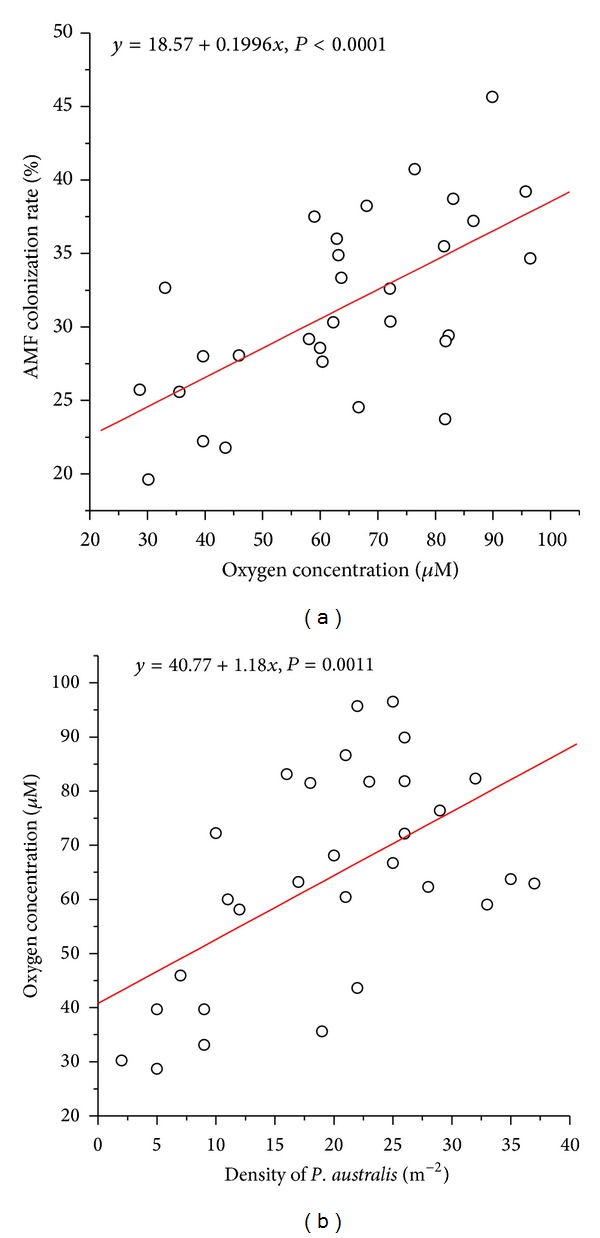
Relationship between the proportion of potential root tissue occupied by mycorrhizal fungus and oxygen concentration (a) and between oxygen concentration and density of *P. australis*.

Field surveys revealed that the proportion of potential root tissue occupied by AMF increased with increasing oxygen concentration (*R* ^2^ = 0.4; *P* < 0.0001; Figure 3(a)), and oxygen concentration was positively correlated with *P. australis *density (*R* ^2^ = 0.32; *P* = 0.0011; Figure 3(b)).

**Figure 4:**
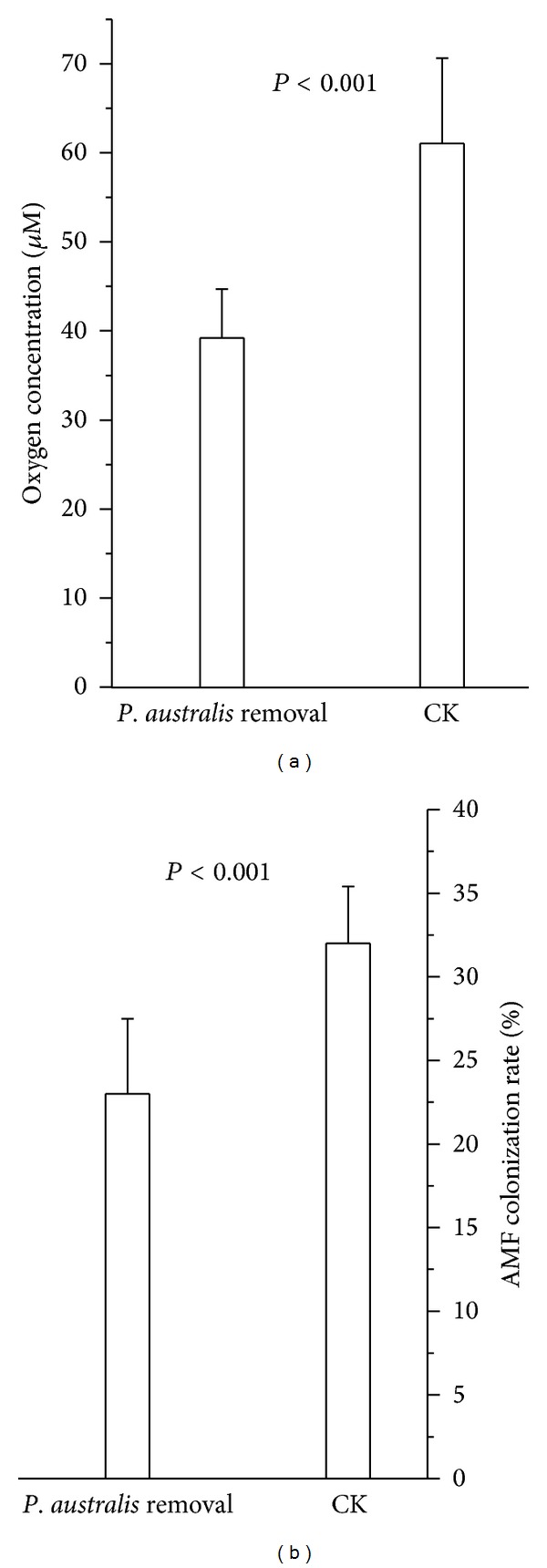
Effect of *P. australis* removal on soil oxygen concentration (a) and AMF colonization on *I. polycephala *(b). CK: control. *P* values are from *t*-test between fungicide application and no fungicide treatments.

In the experiment designed to test the effect of *P. australis *existence on soil oxygenation, removal of *P. australis* led to a decrease in oxygen availability (df = 18, *t* = −6.26, and *P* < 0.001; Figure 4(a)) and AMF colonization on *I. polycephala *(df = 18, *t* = −4.67, and *P* < 0.001; Figure 4(b)).

**Figure 5:**
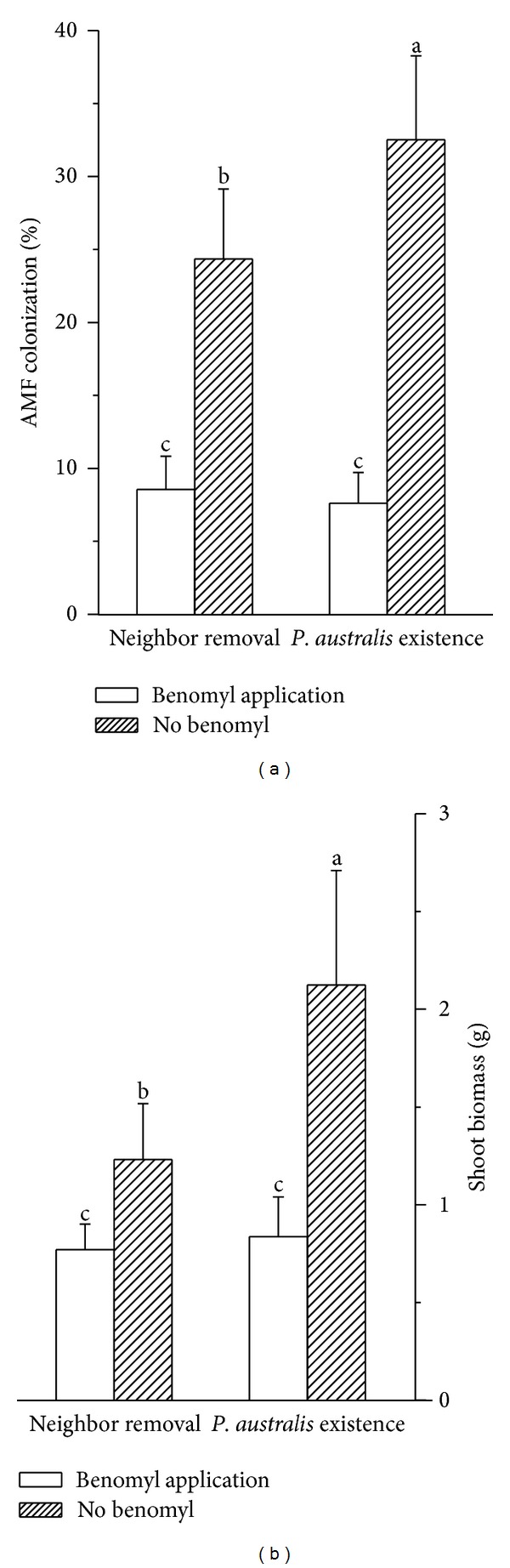
Effect of *P. australis* removal and benomyl application on AMF colonization of *I. polycephala *(a) and shoot biomass (b). Different letters represent significant difference among treatments.

In the experiment designed to examine the hypothesis that *P. australis* affects *I. polycephala* growth by affecting mycorrhizal fungi, benomyl application led to a sharp reduction in the proportion of potential root tissue occupied by AMF (df = 1, *F* = 253.23, and *P* < 0.0001; Figure 5(a)). Neighbor removal also decreased AMF colonization (df = 1, *F* = 7.91, and *P* = 0.0079; Figure 5(a)). The interaction between neighbor removal and benomyl application was significant (df = 1, *F* = 12.71, and *P* = 0.001; Figure 5(a)). Shoot biomass of *I. polycephala *was also decreased by benomyl application (df = 1, *F* = 63.28, and *P* < 0.0001; Figure 5(b)). Neighbor removal also decreased shoot biomass (df = 1, *F* = 19.07, and *P* = 0.0001; Figure 5(b)). Interaction between neighbor removal and benomyl application was significant (df = 1, *F* = 14.18, and *P* ≤ 0.0006; Figure 5(b)).

## 4. Discussion

### 4.1. Effects of Fungicide

Benomyl application suppressed AM fungal colonization. Although some experiments have shown that pathogenic fungi [55] and other soil organisms such as root-feeding nematodes [56] can also be affected by benomyl, others reported that benomyl application had little or no effect on nonmycorrhizal plant and bacterial communities [42]. This is supported by the observation that benomyl application caused no difference in plant growth compared to pasteurized soil with an other soil microflora added back [57]. Because there is no method that only allows the elimination of AMF in a field setting, benomyl application may be one of the best options to suppress AMF in the field compared to other methods [43, 57]. If benomyl affects pathogenic more than mycorrhizal fungi, plant growth should be promoted, not suppressed [57]. We have previously shown that the benomyl effect on *Medicago sativa* L. was mainly due to suppressing mycorrhizal colonization [21, 58]. Here we made soil nutrient analysis with soil enzyme activity and culturable fungal unit measurement. We are confident that our results are actually due to AMF suppression as we found that benomyl application did not have significant effect on soil total nitrogen and mineralizable N, total P and available P, soil urease activity, acid phosphomonoesterase activity, and culturable fungal unit (see supporting information). These results suggest minimal experimental artefacts of benomyl application. Benomyl application led to a much reduced mycorrhizal colonization and decreased plant growth of the three subdominant plant species. Benomyl application did not affect growth of the two dominant plant species, suggesting that the dominant species are less dependent on mycorrhizal colonization than the subdominants.

### 4.2. AM Fungi and Plant Interactions under Waterlogged Conditions

Plant growth may be either dependent or not dependent on mycorrhizal colonization, and AM fungal colonization and variation in AM fungal taxa both may alter interactions among plants in a variety of plant communities [20, 38, 57, 59–62]. To our knowledge, only a few studies have been concerned with the role of AM fungi in plant communities experiencing waterlogging. Daleo et al. [42] reported that mycorrhizal fungi influenced interactions between *Spartina densiflora *and* S. alterniflora *and affected salt-marsh plant community structure. Here we showed that AM fungal colonization is an important contributor to plant growth and neighbor interactions in a plant community experiencing seasonally waterlogged, anaerobic conditions. Three of the five plant species, *K. striata, L. Artemisia*, and *I. polycephala*, showed growth dependence on mycorrhizal colonization. Facilitative neighbor effect on these three species was enhanced by mycorrhizal colonization, while mycorrhizal colonization on *C. canadensis *enhanced the competitive neighbor effect.

Recently studies showed that cooccurring species with different stress tolerance and ecological optima may show differential responses to the same neighbors in a given community [49]. For example, the magnitude of positive neighbor effects among species was negatively correlated with the density of target plant species in an alpine meadow of the Qing-Hai Tibet Plateau [63]. Choler et al. [64] also showed negative neighbor effects on the target plants in the most favorable part of the niche and positive interactions in its most constrained part. Here we show that type (competitive or facilitative) of interspecific neighbor effect was dependent on species when the plant community was waterlogged; neighbor effects were negative or neutral for dominant plant species and facilitative for subdominant plant species.

These species-specific neighbor effects were mainly driven by AMF. In this study we demonstrated that plant species vary in the degree to which they respond to AM fungi and plant neighbors. The dominant species *P. pubescens* and *C. canadensis* exhibited neutral or negative response to AMF and plant neighbors, while the three subdominant species exhibit positive responses to AMF and plant neighbors. The species-specific responsiveness to AMF as a mechanism in which AM fungi influenced plant community structure was first proposed by Bergelson and Crawley [65]. van der Heijden [66] suggested that the number and relative abundance of mycorrhizal-dependent plant species in the species pool can be used to predict how AM fungi affect communities. Here the ability of the three subdominant plant species to coexist with other plant species could therefore be highly dependent on AM fungal symbiosis. In contrast, *C. canadensis* was negatively affected by AM symbiosis and* P. pubescens* would not be directly affected by AM fungi. This high dependence of subdominant plant species on mycorrhiza has been proved to maintain high plant species richness and diversity [67].

It is interesting to note the asymmetry in the delivery of benefit between plant and AM fungi; the two dominant plant species maintain high mycorrhizal colonization but apparently receive little or no growth promotion, while growth and neighbor effects of subdominant plant species were promoted by reduced mycorrhizal colonization. The AM symbiosis may be largely nonspecific, but the extent of plant growth promotion by AM fungi and plant resource allocation to AMF may vary strongly among species [68]. The ecological importance of this interaction can be broadly appreciated; symmetric benefit transfer between plant host and AMF (positive feedback) may cause a decline in species diversity [69], while asymmetric benefit (negative feedback) may contribute to the coexistence of competing plant species [70]. Here the resulting dynamic may contribute to plant species coexistence. The dominant plant species are predicted to support growth and survival of subdominant species by providing mycorrhizal inoculum during the waterlogged season.

While positive interactions among plants have been reported in wetland ecosystems, the mechanisms are mainly explained as protection from abiotic stress [13]. Our surveys and experiments show a strong positive effect of *P. australis* on soil oxygen availability, the major physical factor limiting the development of AMF in wetlands [71], and a positive association between *P. australis* and the proportion of *I. polycephala* roots associated with AM fungi. Field experiments demonstrate that *P. australis* removal leads to large decreases in AMF colonization, confirming that *P. australis* facilitates the presence of AM fungi. We also showed that experimental removal (both by fungicide application and *P. australis* exclusion) of AM fungi leads to large reductions in *I. polycephala* biomass, while, in the benomyl application treatment, neighbor removal did not decrease plant biomass, showing that the primary mechanism by which *P. australis* augments *I. polycephala *plant growth is the facilitation of mycorrhizal association.

Until recently, AMF were considered to be unimportant in wetland communities [41], but our results demonstrate their potential importance in driving plant interactions in a marshland of the Yangtze River. The fact that AM fungi influence neighbor interactions involving subdominant plant species suggests that AMF could be critical in maintaining host plant species richness in this marshland community. However, as only five species were evaluated, establishing the generality of these results requires further substantiation. Further research will also be required to explore the response of AM fungal communities to waterlogging and their feedback to plant interactions and plant community structure and to quantify the relative importance of AM fungi to abiotic factors (e.g., waterlogging) as a driver of community structure and species diversity in marshlands.

## Supplementary Material

Supporting Information: Further details on methods and results of measuring soil total N, mineralizable N, total P and available P, urease activity, acid phosphomonoesterase activity and culturable fungi count.

## Figures and Tables

**Table 1:** Density of the studied species.

Species	Density
*Phragmites australis *	19.3 ± 7.04
*Polygonum pubescens *	25 ± 6.24
*Kummerowia striata *	5 ± 1.33
*Leonurus artemisia *	3.5 ± 0.97
*Ixeris polycephala *	2.3 ± 0.95
*Conyza canadensis *	12.3 ± 4.27

0.50 m × 0.50 m sampling quadrats were randomly arranged in April, before the waterlogging season, and the number of individuals of every species in each quadrat was counted. Data represent means ± SD (*n* = 10 for each species).
