# Supplementary material for: Arbuscular Mycorrhizal Fungal Mediation of Plant-Plant Interactions in a Marshland Plant Community
Source: ScientificWorldJournal. 2014 Feb 12;2014:923610. doi: 10.1155/2014/923610 (PMC3943290; doi:10.1155/2014/923610)
Supplement: Supplementary file 1 — Supporting Information: Further details on methods and results of measuring soil total N, mineralizable N, total P and available P, urease activity, acid phosphomonoesterase activity and culturable fungi count. [file 923610.f1.pdf]

Materials and methods

Soil sampling

Soil samples were collected from ten control and ten benomyl-treated plots randomly selected. From each treatment plot, five samples (5 cm diameter at 5 cm deep) were collected, and composited as one sample. Soil samples were passed through a 4 mm sieve and stored at 4℃ for analysis. Subsamples of soil from each plot were used for determination of soil nitrogen and phosphorus, soil enzyme activity, and cultirable soil fungi count. Gravimetric soil water content of all samples (105℃, 48 h) was determined, and results from microbial analyses are expressed on a per gram oven-dry soil basis.

Soil total N, mineralizable N, total P and available P

Total N concentrations is measured by Kjeldahl procedures (2200 Kjeldahl Auto Distillation). Ammonium and nitrate concentrations were measured with a segmented flow analyzer (SAN plus, Skalar, The Netherlands) after extracted with 1 mol L^{-1} KCl (Oelmann *et al*., 2007). Mineralized N was calculated as the total of ammonium and nitrate (NH_4^+-N + NO_3^--N). Total P and available P concentrations were measured by spectrophotometry (UV-1600 spectrophotometer,Beijing,

Soil enzyme activity

Fresh soil samples for analyzing enzyme activity were sieved (<2.0 mm) before the measurement. Urease activity was measured using the method of Tabatabai (1994) and Bao (2000). Acid phosphomonoesterase activity was determined with the method of Tabatabai and Bremmer (1969). Results were expressed as mg of various products generated by 1.0 kg of dry soil at 37 ℃ per hour. Control tests without soils or substrates were carried out to evaluate the spontaneous or abiotic transformation of substrates (Gianfreda *et al*., 2005).

Culturable fungi count

Fungal count was determined by soil dilution plating. 10 g soil was put into 90 ml

sterile water, stirred vigorously for 30 min, diluted serially, and plated on the Martin medium containing Rose Bengal and 2 ml/L lactic acid. After incubated at 25°C for 4 d, the colony number of soil fungal populations on the plates was counted.

Statistical analysis

T-tests were performed to compare the difference of measured parameters between no-benomyl and benomyl-treated treatments.

Results

Benomyl application did not affect soil nitrogen ($t=0.42$, $p=0.67$, Fig. S1a), soil mineralizable nitrogen ($t=1.36$, $p=0.19$, Fig. S1b), soil total phosphorus ($t=-1.61$, $p= 0.12$, Fig. S1c) and available phosphorus ($t= 0.62$, $p=0.54$, Fig. S1d) significantly.

Both urease activity ($t=1.17$, $p=0.26$, Fig. S2a) and acid phosphomonoesterase activity ($t-1.85$, $p=0.09$, Fig. S2b) were not different significantly benomyl-treated and no-benomyl treatments.

Benomyl application did not affect culturable fungal units significantly ($t= -1.08$, $p= 0.29$, Fig. S3).

Figure legends

Figure S1 Soil total nitrogen, mineralizable nitrogen, total phosphorus and available phosphorus under no-benomyl and benomyl-treated treatments.

Figure S2 Soil urease activity and acid phosphomonoesterase activity under no-benomyl and benomyl-treated treatments.

Figure S3 Soil culturable fungal units under no-benomyl and benomyl-treated treatments.

Fig. S1

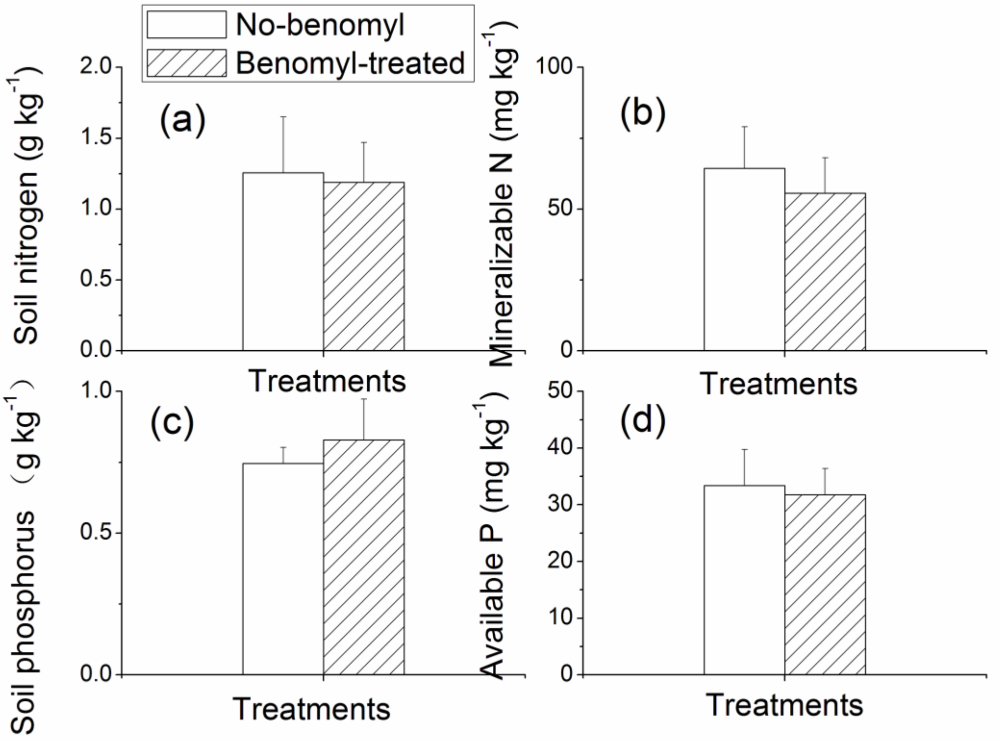
No-benomyl
Benomyl-treated
(a)
Soil nitrogen (g kg-1)
2.0
1.5
1.0
0.5
0.0
Treatments
(b)
Mineralizable N (mg kg-1)
100
50
0
Treatments
(c)
Soil phosphorus （g kg-1）
1.0
0.5
0.0
Treatments
(d)
Available P (mg kg-1)
50
40
30
20
10
0
Treatments

Fig. S2

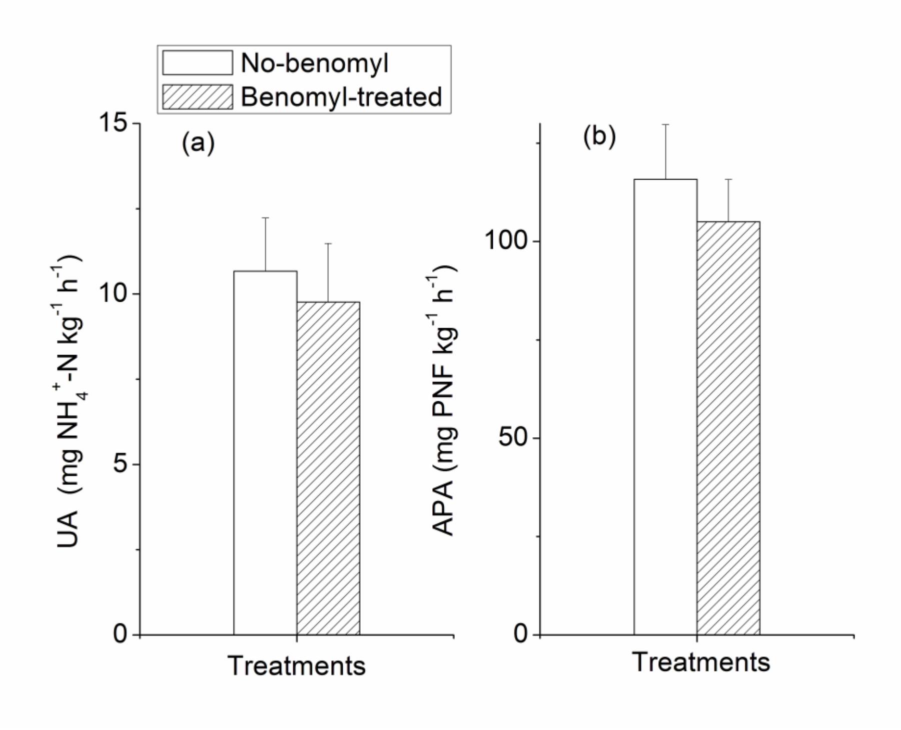

No-benomyl
Benomyl-treated
(a)
UA (mg NH4+-N kg-1 h-1)
15
10
5
0
Treatments
(b)
APA (mg PNF kg-1 h-1)
100
50
0
Treatments

Fig. S3

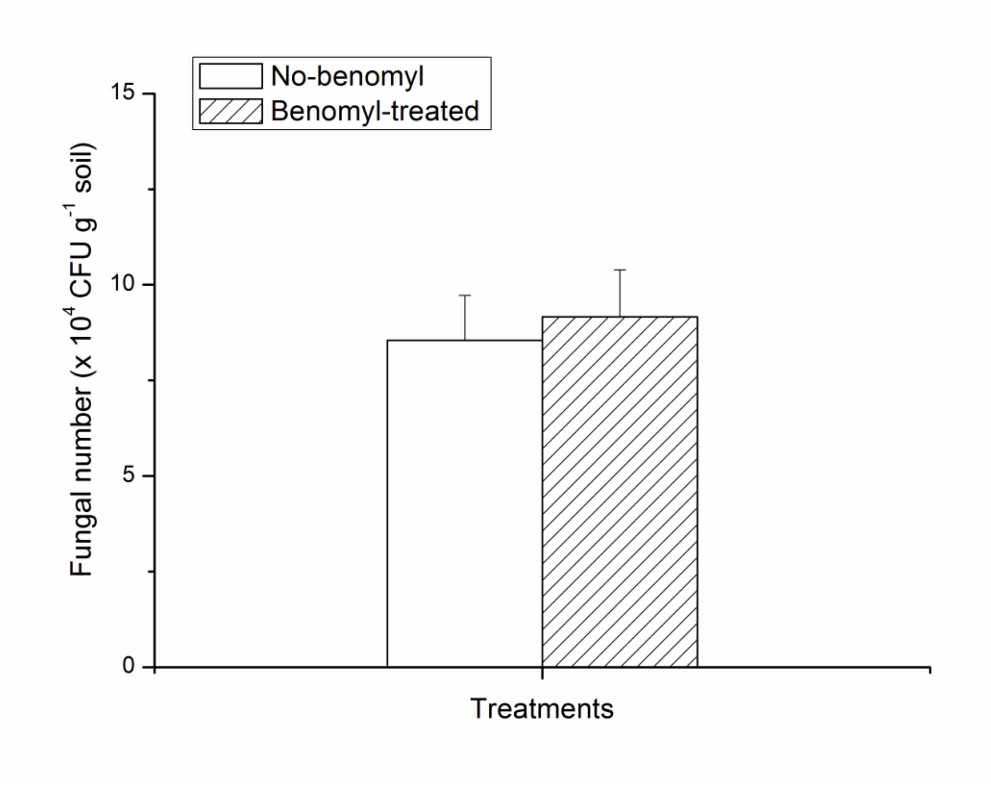

Reference

Oelmann Y, Wilcke W, Temperton VM, Buchmann N, Roscher C, Schumacher J, Schulze ED, Weisser WW. 2007. Soil, Plant Nitrogen Pools as Related to Plant Diversity in an Experimental Grassland. *Soil Science Society of America Journal* 71: 720–729.

Bao SD. 2000. Method of soil chemistry analysis. Beijing: Chinese Agriculture Science and Technology Publishers, pp. 373–375.

Tabatabai MA. 1994. Soil enzymes. In: Weaver, R.W., Angle, J.S., Bottomley, (Eds.), Method of Soil Analysis. Part 2. Microbiological and Biochemical Properties. SSSA Book Series, vol. 5. Soil Sci Soc Am, Madison, WI, pp. 775–833.

Tabatabai MA, Bremmer JM. 1969. Use of p-nitrophenyl phosphate for assay of soil phosphatase activity. *Soil Biology and Biochemistry* 1: 301–307.

Gianfreda L, Rao MA, Piotrowska A, Palumbo G, Colombo C. 2005. Soil enzyme activities as affected by anthropogenic alterations: intensive agricultural practices and organic pollution. *Science of the Total Environment* 341: 265–279.
